# Measuring Workload Among Family Medicine Health Professionals During the COVID-19 Pandemic

**DOI:** 10.7759/cureus.66769

**Published:** 2024-08-13

**Authors:** Ronscardy F Mondesir, Yaqoub Yusuf, Hayley Dykhoff, Alexandra Watral, Robert Peck, Steven Rosas, Heather Logslett, Renaldo Blocker

**Affiliations:** 1 Department of Medical and Population Health Sciences Research, Florida International University, Herbert Wertheim College of Medicine, Miami, USA; 2 Department of Family Medicine, Mayo Clinic Health System, Rochester, USA; 3 Department of Family Medicine, Robert D. and Patricia E. Kern Center for the Science of Health Care Delivery, Mayo Clinic, Rochester, USA

**Keywords:** healthcare professional, coronavirus disease 2019 (covid-19), healthcare provider, covid-19, excessive workload

## Abstract

The global COVID-19 pandemic presented a period of considerable stress for healthcare professionals on a global scale. The strain on healthcare facilities nationwide has resulted in profound implications for the well-being of numerous healthcare practitioners. A heightened demand for extended working hours emerged, potentially amplifying the workload for these professionals. This study aims to scrutinize the workload levels experienced by healthcare professionals specializing in family medicine at a tertiary medical center. Our findings reveal a persistent escalation in workload over the course of the study. Notably, the overall mean workload index score exhibited a substantial increase from phase one to phase two (48.07 compared to 66.36). Recognizing the impact of workload variations according to professional roles is crucial for devising effective solutions. Consequently, comprehending the nuances of workload distribution among healthcare professionals is imperative for the successful implementation of targeted interventions.

## Introduction

An increase in workload and clinical burden can negatively impact the effective delivery of clinical care and lead to medical errors in an already stressful environment [1]. As the workload intensifies for healthcare providers (HCPs), the occurrence of medical errors increases significantly. These errors range from misdiagnoses and poor patient history documentation to inaccuracies in health records and incorrect medication prescriptions [2-4].

Previous research has shown that a high workload burden possesses negative ramifications on surgeons and surgical staff as surgeons conduct long hours of surgical procedures that impose severe mental and physical distress [5,6]. Such severe levels of distress can lead to surgical errors, poor awareness in the operating room, lack of communication among the surgical team, and ultimately impact the health outcomes of patients undergoing complex lifesaving procedures [7,8]. Additional research has also highlighted the potential effect of workload burden on reducing the positive ambience of a working environment, which is substantial for reducing employee turnover and promoting engagement and productivity [9]. Among healthcare professionals, burnout is widely prevalent among family physicians practicing in the primary care workforce due to increasing workloads that impose severe mental and physical strain [10]. According to the American Academy of Family Physicians, common driving factors of burnout among family physicians include high workload, high volume of paperwork, the use of electronic health records, frustration with referral networks, and feeling undervalued [11].

Limited research examines clinicians' workloads during the pandemic. This paper will analyze workloads during two high-peak time periods in 2020. Understanding workloads during these periods will shed light on the impact of COVID-19 on the medical community, particularly in terms of increased burnout. Hence, this research aims to identify the specific challenges faced by family medicine professionals and provide insights into how these challenges can be mitigated in future healthcare crises.

We hypothesized that workload levels during the pandemic were significantly higher, leading to increased stress and burnout among healthcare professionals. Additionally, this paper will provide valuable insights for healthcare administrators and policymakers to develop strategies that can better support healthcare professionals during times of crisis, ensuring the delivery of safe and effective patient care while maintaining a positive work environment for healthcare staff.

## Materials and methods

Our research was conducted in two seven-week phases in 2020: phase one (PI) spanned from mid-April to May, whereas phase two (PII) extended from late September to mid-November. Invitations for study participation were emailed to healthcare personnel across three hospitals within a healthcare system in Northwest Wisconsin. In PI, 51 healthcare staff, comprising 27 nursing staff, seven advanced practice providers (APPs), and 17 physicians, were invited. Due to staffing changes, PII invited 53 healthcare staff (30 nursing staff, seven APPs, and 16 physicians), with a 94% overlap from PI. Those healthcare staff responding to the invitation provided consent and became participants in the study phase.

Throughout each day of the study, participants received a consistent survey prompting reflections on their most recent shift. This survey, derived from the NASA task load index (NASA-TLX), encompassed seven subscale items gauging various workload aspects (Table 1). Participants rated each subscale on a Likert scale (1-20), with 20 indicating high workload and 1 indicating low workload. Subscale scores were aggregated for a composite score (potential range: 7-140). Statistical tests for NASA-TLX scores comparing healthcare roles were executed using general linearized modeling, adjusting for participant ID. Tukey's honest significant difference tests were applied to identify statistically significant differences in roles, considering multiple comparisons.

**Table 1:** NASA-TLX subscale questions from the survey NASA-TLX: NASA task load index

NASA-TLX subscale	Question in survey
Mental demand	How mentally demanding was your shift?
Physical demand	How physically demanding was your shift?
Temporal demand	How hurried or rushed was the pace of your shift?
Effort	How hard did you have to work to accomplish your level of performance?
Performance	How successful were you in accomplishing everything you intended to do during your shift?
Frustration	How irritated, stressed, and/or annoyed were you during your shift?
Distraction	How distracted did you feel during your shift?

The survey also featured two open-ended questions allowing participants to recount their shift qualitatively: "What went well today?" and "What specific worries do you have?" Responses were categorized nonexclusively based on themes, with a single response potentially falling under multiple themes.

## Results

Phase 1

**Figure 1:**
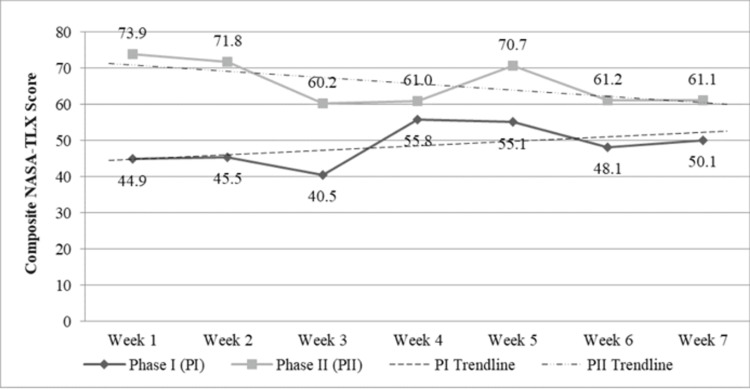
Overall mean composite NASA-TLX scores over weeks 1-7 in phases I and II NASA-TLX: NASA task load index

We received responses from 33 unique individuals (66.0% of invited staff; 14 nursing staff, six APPs, and 13 physicians) and 479 overall survey responses in PI, with 293 (61.2%) of them containing at least one NASA-TLX subscale response, which we could use in the analysis. The overall mean composite NASA-TLX score was 48.07 (SD = 24.70) and tended to increase over the phase period (Figure 1). Of the three roles, the nursing staff had the highest overall mean composite score of 68.73. Physicians had an overall mean composite score of 43.02, and APPs responded similarly, with a mean of 40.32.

There was variability among the roles in terms of mean subscale scores, all of which are displayed in Table 2. Nursing staff consistently indicated a higher workload, with 6 out of 7 (6/7) subscales having a significantly higher workload than both APPs and physicians. Notably, the only subscale on which nursing staff did not score higher than the other roles was performance (where one indicates complete success and 20 indicates complete failure).

**Table 2:** Phase 1 mean scores on each subscale and overall on the NASA-TLX organized by profession ^a,b,c^The test results of the ANOVA followed by Tukey's multiple comparison test Means within a row without a common superscript indicate significant differences at p <0.05. NASA-TLX: NASA task load index; APP: advanced practice provider

Scale (score range)	Role		
	APP	Nursing staff	Physician
Composite (7-140)	38.16^a^	67.10^b^	43.06^a^
Mental demand (1-20)	7.44^a^	11.64^b^	8.08^a^
Physical demand (1-20)	3.54^a^	6.74^b^	3.41^b^
Temporal demand (1-20)	5.72^a^	10.76^b^	6.14^a^
Effort (1-20)	7.52^a^	11.47^b^	8.41^a^
Performance (1-20)	4.55^a^	8.43^b^	8.45^b^
Frustration (1-20)	6.11^a^	10.77^b^	5.01^a^
Distraction (1-20)	5.12^a^	9.12^b^	6.58^c^

Of the 479 surveys received, 139 surveys (29.0%) responded to the “What went well today?” question. The most common response themes were good team collaboration (27.3%), administrative successes (e.g., having a full schedule; 22.3%), and having a smooth day (21.6%). Participants also mentioned having technological success when conducting video visits with patients and virtual meetings with colleagues (15.1%).

Seventy-five surveys (15.7%) responded to the “What specific worries do you have?” question. Participants commonly identified being worried about personal financial changes (30.7%), particularly with regard to some staff being in danger of imminent furlough or reduced working hours. Other common worries included acclimating to new changes in workflow (e.g., changes to emergency room protocols; 32.0%) and the possibility of COVID-19 infection for themselves or family members (20.0%).

Phase 2

In PII, 33 unique individuals responded to at least one survey (62.3% of invited staff; 18 nursing staff, three APPs, and 12 physicians). We received a lower number of overall survey responses compared to PI (N = 298), but a larger portion of them contained at least one NASA-TLX subscale response (N = 252, 84.6%). The overall mean composite NASA-TLX score was higher during PII than PI: 66.36 (SD = 25.38) and, unlike PI, decreased over the seven-week period (Figure 1). Nursing staff still had the highest overall mean composite score of 70.76, closely followed by APPs with a mean of 67.40 and physicians with a mean of 57.27.

APPs responded with higher workload scores in PII, whereas mean scores for nursing staff and physicians remained closer to PI values. In PII, APPs displayed subscale means that are more similar to those of the nursing staff than the physicians. In 5 out of 7 subscales, nursing staff and APPs reported a significantly higher workload than physicians (Table 3).

**Table 3:** Phase 2 mean scores on each subscale and overall on the NASA-TLX organized by profession ^a,b,c^The test results of the ANOVA followed by Tukey's multiple comparison test Means within a row without a common superscript indicate significant differences at p <0.05 NASA-TLX: NASA task load index; APP: advanced practice provider

Scale (score range)	Role		
	APP	Nursing staff	Physician
Composite (7-140)	67.20^a^	72.07^a^	57.69^b^
Mental demand (1-20)	13.02^a^	11.89^a^	9.57^b^
Physical demand (1-20)	8.39^a^	9.74^b^	5.08^c^
Temporal demand (1-20)	10.16^a^	11.77^a^	9.35^b^
Effort (1-20)	12.57^a^	12.48^a^	9.95^b^
Performance (1-20)	4.23^a^	7.14^b^	6.72^b^
Frustration (1-20)	10.52^a^	10.02^a^	7.81^b^
Distraction (1-20)	8.32^a^	9.06^a^	8.90^a^

Of the 298 surveys received in PII, “What went well today?” was answered in 109 of them (36.6%), which was more frequently than in PI. Participants' most common positive themes continued to be good teamwork and collaboration (43.1%) and administrative successes (e.g., having a full schedule; 31.2%), and these themes appeared more frequently in responses than in PI. A new theme emerged: acknowledgment or appreciation of specific coworkers (e.g., “Go team!” 12.8%).

Ninety-two surveys (30.1%) contained responses to the question about participants' worries. Most responses discussed concerns about the contemporaneous COVID-19 infection surge (37.0%) and inadequate staffing to handle it (33.7%). Interestingly, the response theme of COVID-19 infection in their personal lives was nearly identical during PII as it was during PI (19.6% in PII vs. 20.0% in PI). Because the majority of PII occurred in October 2020, many responses also mentioned the U.S. political climate and upcoming elections (19.6%).

## Discussion

Overall, we saw an increase in perceived workload from PI to PII. We initially found these results surprising because we had anticipated the opposite; we thought healthcare staff would feel better equipped/prepared six months after PI. However, PII brought a potent contextual factor to consider: an enormous increase in the number of new COVID-19 cases in the United States and worldwide [12].

When comparing the means of overall composite NASA-TLX scores by roles, we found nurses to generally have a higher overall score than other roles, which is consistent with current literature that provides evidence of higher workload for nurses during the pandemic because of substantially more time spent providing individualized care to COVID patients [13].

The noticeable trend of higher workload for nurses in phase I can also be associated with the increased demand for nurses, reduced staffing, increased overtime, and inadequate supply of nurses [14,15]. As a result, the shortage of nurses results in a higher workload for the nurses who remain on the job [15] and ultimately induces absenteeism, turnover, poor job performance, and threatens patient care and the effectiveness of organizations [14,15]. In PI, nurses scored slightly lower than the other roles in one of the subscales: performance, a trend that could have resulted in group dynamics or resources available to help the nurses be more efficient.

Several limitations should be considered when interpreting the results of this study. First, the sample size may have impacted our ability to detect significant effects or associations. Inadequate sample sizes can lead to underpowered studies, potentially failing to identify true treatment effects or relationships between variables [16,17]. Second, the lack of a comparison group, particularly in the context of pre- and post-COVID-19 periods, limits our ability to draw causal inferences and assess the specific impact of the pandemic on our outcomes [17]. Third, the generalizability of our findings may be limited due to the specific characteristics of our study population and setting. Results obtained from a single institution or region may not represent broader populations or healthcare systems [18]. Finally, potential biases, such as selection bias, recall bias, or observer bias, could have influenced our results. These biases can lead to systematic errors in data collection, analysis, or interpretation, potentially affecting the validity of our conclusions [18].

These limitations could have been addressed through studies that focused on employing larger sample sizes, including appropriate comparison groups, expanding the study to multiple sites or regions, and implementing strategies to minimize potential biases during the COVID-19 pandemic. Alkhamees et al. conducted a systematic review and meta-analysis to examine the impact of COVID-19 on burnout. A total of 30 studies included a large sample size, and they found that burnout rates among physicians during COVID-19 ranged widely from 6.0% to 99.8%, which is much higher than pre-pandemic times. Although our study had a small sample size, our results correlate with this large meta-analysis, concluding that the COVID-19 pandemic contributed to the increase in burnout among HCPs [19].

Several strengths of this study enhance its contributions to the existing literature. First, it provides real-time data on healthcare workers' perceived workload during different phases of the COVID-19 pandemic, offering valuable insights into the evolving challenges faced by HCPs. Second, by utilizing the NASA-TLX score to quantify workload across different roles, the study ensures a standardized and comprehensive assessment of workload dimensions. Third, the focus on physicians, APPs, and nurses, who have been disproportionately affected by the pandemic, highlights critical issues related to staffing and workload management. Additionally, the study's findings emphasize the need for robust health policies and workforce management strategies, thereby contributing to the discourse on improving healthcare systems' resilience during crises.

Implications for policy, practice, and research

While the impact of the COVID-19 pandemic is global, the workload and clinical burden experienced by U.S. HCPs may have been preventable with robust health policies that plan for an unexpected increase in demand for healthcare services. The increased workload that our research detected may have negatively impacted those who contracted the virus and individuals who needed to seek care for other emergent medical conditions since burnout can reduce the quality of care. To prevent such a heavy strain on our medical providers in the future, swift and actionable measures are a prerequisite to addressing underlying weaknesses in our health system. For instance, a mandatory hour cap can be implemented during health care emergencies while allowing nursing and medical students near to finishing their degrees to contribute to increasing health needs.

The COVID-19 pandemic has highlighted the need for robust health policies to manage unexpected surges in healthcare demand and protect healthcare workers from burnout. While research on hourly caps, specifically during pandemics, is limited, studies on work-hour restrictions in normal circumstances provide insights into their potential benefits.

A systematic review by Moonesinghe et al. examined the impact of resident duty hour restrictions on patient safety and resident well-being. The review found that reducing weekly work hours was associated with decreased medication errors and improved patient mortality and length of stay in intensive care [20]. Similarly, a study by Landrigan et al. on the effects of reducing intern work hours in intensive care units reported that interns made substantially more serious medical errors when they worked frequent shifts of 24 hours or more than when they worked shorter shifts and concluded that eliminating extended work shifts and reducing the number of hours interns work per week can reduce serious medical errors in the intensive care unit [21]. These findings suggest that limiting work hours could potentially decrease workload and improve patient safety during high-stress periods like pandemics to reduce burnout rate.

However, the implementation of hour caps is not without challenges. Hospitals may need to hire additional staff or restructure programs to comply with hour caps, which can have significant financial implications. Most importantly, some studies have shown that mandating hour caps may increase the workload for healthcare workers as they may be subject to completing the same amount of work with less available time, which can negatively impact patient care [22]. Therefore, any policy implementing hour caps during health emergencies should carefully balance workload reduction with maintaining quality of care. Future research should specifically examine the effects of implementing hour caps during pandemic situations. Such studies could provide valuable insights into optimizing healthcare workforce management during crises while ensuring provider well-being and patient safety.

## Conclusions

Our study found an unexpected increase in perceived workload from PI to PII among healthcare staff, with a particularly notable rise during PII coinciding with a surge in COVID-19 cases globally. This finding is surprising because we had anticipated decreased perceived workload over time as HCPs adapted to pandemic-related challenges. The increased workload among nurses, evidenced by higher overall NASA-TLX scores compared to other roles, aligns with existing literature on the disproportionate burden nurses face during the pandemic due to the demands of individualized care for COVID-19 patients. The results also highlighted the severe consequences of nurse shortages, including increased overtime, reduced staffing, and the resultant higher workloads, which contribute to absenteeism, turnover, and compromised patient care.

These findings contribute to the literature by highlighting the significant impact of the COVID-19 pandemic on healthcare workloads. The staggering aspect of these results lies in the documented escalation of workload despite anticipated improvements in preparedness over time. This highlights the necessity for robust health policies to manage healthcare demands during emergencies, such as implementing mandatory work-hour caps. Evidence from related studies indicates that reducing work hours can decrease medical errors and improve patient safety, suggesting potential benefits for mitigating burnout during crises.

Furthermore, our study's findings call for future research on the effectiveness of hour caps during pandemics and the need for strategic workforce management to balance workload reduction with maintaining care quality. These insights are crucial for developing policies that protect healthcare workers' well-being and ensure patient safety during high-stress periods like pandemics.
